# A propensity score-matched study including 250,000 patients with Factor V Leiden shows significantly increased mortality in comparison with individuals without thrombophilia

**DOI:** 10.1016/j.puhip.2026.100751

**Published:** 2026-02-16

**Authors:** Moritz Hertel, Max Heiland, Susanne Nahles, Saskia Preissner, Robert Preissner

**Affiliations:** a Department Oral and Maxillofacial Surgery, Charité – Universitätsmedizin Berlin, Corporate Member of Freie Universität Berlin, Humboldt-Universität zu Berlin, Augustenburger Platz 1, 13353, Berlin, Germany; b Institute of Physiology and Science-IT, Charité – Universitätsmedizin Berlin, Corporate Member of Freie Universität Berlin, Humboldt-Universität zu Berlin, Philippstrasse 12, 10115, Berlin, Germany

**Keywords:** Factor V Leiden, Thrombophilia, Venous thrombosis, All-cause mortality, Real-world data

## Abstract

**Background:**

Factor V Leiden (FVL) is a common inherited thrombophilia that increases the risk of venous thromboembolism (VTE). However, its effect on all-cause mortality remains uncertain, and previous studies yielded conflicting results.

**Objectives:**

To assess the association between FVL and all-cause mortality using a large international database, including age- and sex-stratified analyses.

**Study design:**

Retrospective propensity score-matched cohort study using real-world electronic health records.

**Methods:**

We analyzed electronic health records from the TriNetX Global Health Research Network, comparing 241,572 patients with FVL to 20,779,115 patients without thrombophilia. One-to-one propensity score matching for age was applied. Incidences of VTE and all-cause mortality were compared over a 19-year follow-up. Subgroup analyses were performed for female patients and three age categories: <20, 20–50, and >50 years.

**Results:**

FVL was significantly associated with both VTE (OR: 9.325; 95% CI: 9.109–9.546) and all-cause mortality (OR: 1.423; 95% CI: 1.396–1.451). The mortality risk was particularly elevated in young females (<20 years: OR: 9.681; 20–50 years: OR: 2.345) and attenuated in older females (>50 years: OR: 1.432). Kaplan-Meier analyses showed significantly reduced survival probabilities in FVL-positive patients across all subgroups.

**Conclusions:**

This large-scale real-world study indicates that FVL is not only a major risk factor for thrombosis but also associated with increased all-cause mortality—particularly among young women. These findings may have implications for screening and risk stratification in selected populations.

## 1 Introduction

The Factor V Leiden mutation is one of the most frequent genetic causes of thrombophilia. This point mutation in the Factor V gene leads to resistance against activated protein C, creating a procoagulant state that significantly increases the risk of venous thromboembolism (VTE) [1,2]. While the link between FVL and VTE is well established, its impact on all-cause and cardiovascular mortality remains debated [3].

Several epidemiological studies have explored this question. Roest et al. (1999) investigated older women and found no association between FVL and cardiovascular mortality [4]. Similarly, Heijmans et al. (1998) reported no increase in all-cause or cause-specific mortality among elderly individuals with the mutation [5]. Both studies concluded that heterozygous FVL does not affect mortality in their respective populations [4,5]. In contrast, homozygous FVL has been described as a rare but severe risk factor for pulmonary embolism with potentially fatal outcomes [6]. Animal studies have also shown decreased survival in heterozygous and homozygous mutation carriers [7].

Given the apparent paradox that FVL significantly increases thrombosis risk without clearly affecting mortality, we hypothesized that carriers might indeed exhibit increased all-cause mortality. The aim of this study was to investigate this association using real-world data from an international health records network, including age- and sex-stratified subcohorts.

Our findings may provide new insights into the clinical management of FVL carriers and contribute to optimized screening and preventive strategies. By examining potential modifiers such as age and sex, we aim to clarify whether FVL represents an independent risk factor for mortality or acts in combination with other variables.

## 2 Methods

### 2.1 Study design

This study was designed as a retrospective cohort analysis based on anonymized electronic health records. Propensity score matching was applied to compare outcomes between patients with and without Factor V Leiden mutation.

### 2.2 Data source, cohort definition, and inclusion criteria

This retrospective cohort study used data from the TriNetX Global Health Research Network (Cambridge, MA, USA), which includes anonymized electronic health records from over 275 million patients across 19 countries. We identified all patients who underwent genetic testing for Factor V Leiden (FVL) and had at least one inpatient encounter. Patients with a positive FVL result were assigned to Cohort I; those with a documented negative result formed Cohort II. Only patients with continuous medical records covering a 19-year period (6935 days) were included. This threshold was chosen as a pragmatic balance between sufficient long-term follow-up for detecting rare events such as mortality and the availability of comprehensive longitudinal data across the TriNetX network.

### 2.3 Index event and time frame

The index date was defined as the FVL test date or the nearest inpatient encounter within ±30 days. Patient age was determined as the age at this index date and used for all subsequent analyses, including matching and stratification. Follow-up was measured from the index date to last documented contact or death. Right and interval censoring were applied.

### 2.4 Outcome measures

The primary outcome was all-cause mortality. The secondary outcome was venous thrombosis, identified via ICD-10 code I82.

### 2.5 Propensity score matching

We performed 1:1 propensity score matching based on patient age using nearest-neighbor greedy matching with a caliper of 0.25 standard deviations. Matching was repeated for each sex- and age-stratified subcohort. Baseline characteristics before and after matching are shown in Table 1.

**Table 1:** Characteristics of cohorts I (Factor V Leiden mutation) and II (no Factor V Leiden mutation) before and after propensity score, matching for age at the time of the index event.

Cohort	Factor V mutation	Mean age (years) and standard deviation	Number of patients	p	Standardized difference
Before propensity score matching					
I	+	49.8 ± 19.9	241,572	<0.001	0.184
II	-	45.7 ± 24.7	20,779,115		
After propensity score matching					
I	+	49.8 ± 19.9	241,572	1	<0.001
II	-	49.8 ± 19.9	241,572		

### 2.6 Statistical analysis

Risk ratios (RR), odds ratios (OR), and hazard ratios (HR) with 95% confidence intervals were calculated. Kaplan-Meier curves were generated and compared using the log-rank test. All analyses were conducted using the TriNetX analytics platform. A p-value <0.05 was considered statistically significant.

### 2.7 Subgroup and sensitivity analyses

As a secondary analysis, we conducted age-stratified survival analyses within the female subcohort to explore potential age-related differences in the association between Factor V Leiden and all-cause mortality. As a sensitivity analysis, we additionally performed the same age-stratified analysis in the male subcohort to test whether any observed associations in women were sex-specific.

## 3 Results

### 3.1 Cohort characteristics

Data were extracted from TriNetX on November 19, 2024. Sixty healthcare organizations contributed eligible data. Cohort I (FVL-positive) included 241,572 patients with a mean age of 49.8 ± 19.9 years; Cohort II (FVL-negative) comprised 20,779,115 patients with a mean age of 45.7 ± 24.7 years.

After 1:1 propensity score matching for age, both cohorts consisted of 241,572 patients (mean age: 49.8 years; p = 1.000). Baseline characteristics are shown in Table 1. A detailed overview of the patient selection process is provided in Supplementary Fig. S1 (modified CONSORT diagram).

### 3.2 Risk analysis

In the matched cohorts, 27,296 FVL-positive patients (11.3%) and 19,843 FVL-negative patients (8.2%) died during follow-up (p < 0.001). The OR for mortality was 1.423 (95% CI: 1.396–1.451). Venous thrombosis occurred in 61,588 FVL-positive patients (25.5%) vs. 8551 in the control group (3.5%), with an OR of 9.325 (95% CI: 9.109–9.546). Results are summarized in Table 2. Additional risk tables are provided in Supplementary Table S1–S2.

**Table 2:** Analysis of the risk of death among patients with Factor V Leiden mutation (cohort I) and individuals without Factor V Leiden mutation (cohort II).

Risk of death analysis							
Cohort	Factor V mutation	Patients in cohort	Patients with outcome	Risk	p	Risk ratio (95% CI)	Odds ratio (95% CI)
I	+	241,572	27,296	11.3%	<0.001	1.376 (1.352-1.400)	1.423 (1.396-1.451)
II	-	241,572	19,843	8.2%			

### 3.3 Kaplan-Meier survival analysis

**Fig. 1:**
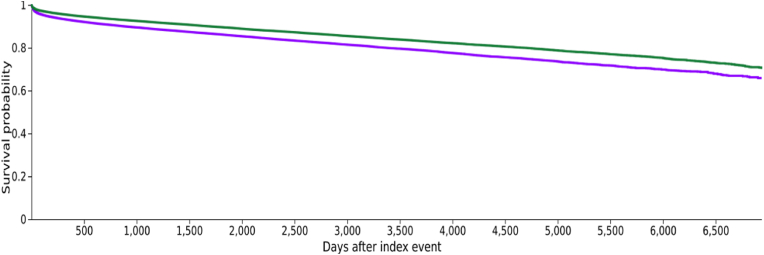
Kaplan-Meier survival analysis of individuals positively tested for Factor V Leiden mutation (purple line) and subjects without Factor V mutation (green line). The survival probability at the end of the time window of 6.935 days was significantly different (65.90% vs. 70.69%; p < 0.001; Log-Rank test).

Kaplan-Meier analysis showed significantly lower survival in FVL-positive individuals (65.90%) vs. FVL-negative controls (70.69%) over 19 years (p < 0.001). The hazard ratio was 1.365 (95% CI: 1.341–1.391). The survival curve is shown in Fig. 1.

### 3.4 Subcohort analysis

#### 3.4.1 Female subcohort

To further explore sex-specific differences, we analyzed female patients separately. After age matching, subcohort III (FVL-positive) and IV (FVL-negative) each included 143,334 women (mean age: 46.5 years; p = 1.000). During follow-up, 13,042 FVL-positive and 8973 FVL-negative women died, corresponding to mortality risks of 9.1% vs. 6.3% (OR: 1.499; 95% CI: 1.458–1.541). Thrombosis occurred in 32,587 FVL-positive and 4151 FVL-negative women (22.7% vs. 2.9%; OR: 9.866; 95% CI: 9.543–10.200). Survival analysis showed significantly lower probability in the FVL-positive group (71.25% vs. 77.91%; HR: 1.461; 95% CI: 1.422–1.501). Detailed data are provided in Supplementary Table S3 and Supplementary Fig. S2. In contrast to the findings observed in the female subcohort, age-stratified survival analyses in the male subcohort revealed no significant differences in all-cause mortality between individuals with and without FVL across all age groups.

#### 3.4.2 Age-stratified female subcohorts

We then stratified female patients by age: <20 years (V/VI), 20–50 years (VII/VIII), and >50 years (IX/X). After matching, each FVL-positive subcohort showed significantly higher mortality than its control:

• <20 years: OR = 9.681 (95% CI: 6.092–15.385)
• 20–50 years: OR = 2.345 (95% CI: 2.148–2.561)
• >50 years: OR = 1.432 (95% CI: 1.390–1.476)

Similarly, thrombosis risk was consistently elevated:

• <20 years: OR = 64.293 (95% CI: 73.077–111.486)
• 20–50 years: OR = 13.915 (95% CI: 12.998–14.897)
• >50 years: OR = 8.854 (95% CI: 8.517–9.204

Survival probabilities were also lower in all age groups:

• <20 years: HR = 7.474 (95% CI: 4.711–11.859)
• 20–50 years: HR = 2.182 (95% CI: 1.999–2.381)
• >50 years: HR = 1.352 (95% CI: 1.315–1.391)

These results are summarized in Supplementary Tables S4–S6 and Supplementary Figures S3–S5.

## 4 Discussion

This large real-world cohort study demonstrates that carriers of the Factor V Leiden (FVL) mutation have a significantly increased risk of all-cause mortality compared to individuals without thrombophilia. In addition, the mutation is strongly associated with venous thrombosis, confirming prior findings. The mortality risk was particularly elevated among younger women.

Previous studies have reported inconsistent findings regarding mortality in FVL carriers. Roest et al. (1999) found no increase in cardiovascular mortality among postmenopausal women [4], and Heijmans et al. (1998) observed no effect on overall mortality in the elderly [5]. Similarly, a family-based Dutch study by Hille et al. (1997) reported no increase in standardized mortality ratios [8]. In contrast, our study included a broader and younger population, allowing for age-stratified analysis with greater statistical power.

The age-specific findings in women are particularly notable. Young female carriers (<20 years) showed a nearly tenfold increase in mortality. As a sensitivity analysis, we performed the same age-stratified survival analyses in a male subcohort. In contrast to the female findings, these analyses revealed no significant differences in all-cause mortality between FVL-positive and FVL-negative males across all age groups. Although the dataset did not include medication data, this result may reflect the known interaction between FVL and estrogen-containing contraceptives, which has been shown to dramatically increase the risk of thrombotic events [9]. This potential mechanism warrants further investigation.

Our study benefits from a very large, international dataset and the ability to perform subgroup analyses in populations that were underrepresented in earlier research. However, several limitations must be acknowledged. First, this was a retrospective analysis based on electronic health records, with possible residual confounding. Propensity score matching was limited to age; other unmeasured variables such as comorbidities, socioeconomic factors, or medication use could have influenced the results. Second, patients were included based on FVL testing, which is typically performed in individuals with suspected thrombophilia. This introduces potential selection bias, as the control group may include undiagnosed FVL carriers. Finally, the use of ICD-10 code I82 to define thrombosis does not allow for differentiation between acute and chronic events. Moreover, since only individuals with documented inpatient encounters were included, and follow-up began at the time of genetic testing, we may have missed outcome events that occurred prior to the index encounter. Moreover, although the TriNetX network includes patient data from multiple countries, we were unable to adjust for ethnicity due to missing or inconsistently coded demographic information. Given known differences in FVL prevalence and thrombotic risk profiles between ethnic groups, this represents a potential limitation in terms of generalizability and external validity. Additionally, although multivariable adjustment models could theoretically increase statistical power by retaining the full cohort, the limited availability and consistency of relevant covariates within TriNetX constrained this approach.

Despite these limitations, our findings suggest that FVL may be associated with increased mortality risk in specific populations, particularly young women. Further prospective studies are needed to confirm these results and investigate the role of hormonal and genetic interactions. From a public health perspective, targeted screening and preventive strategies in selected subgroups—such as young women with a positive family history—may be warranted.

### 4.1 *Conclusion*

In this large, international real-world study, the Factor V Leiden mutation was associated not only with a markedly increased risk of venous thrombosis but also with a significant elevation in all-cause mortality. The effect was most pronounced among young female carriers, suggesting a possible interaction between genetic and hormonal factors. These findings may inform future risk stratification strategies and support the case for targeted screening in specific high-risk populations. Further prospective studies are needed to validate these observations and assess their clinical and public health implications.

## Ethics statement

The study used de-identified data from the TriNetX Global Health Research Network. According to the local regulations (German Research Guidelines and §15 of the Berlin State Hospital Act), the use of fully anonymized retrospective data does not require approval by an institutional ethics committee. Therefore, ethical approval was waived by the Ethics Committee of Charité – Universitätsmedizin Berlin. The requirement for informed consent was waived as all data were anonymized at the source.

All methods were carried out by relevant guidelines and regulations. All HCOs from which data were reported to TriNetX obtained informed consent in written form from all patients and their legal guardians. TriNetX complies with the Health Insurance Portability and Accountability Act (HIPAA), the US federal law that protects healthcare data's privacy and security. TriNetX is certified to the ISO 27001:2013 standard and maintains an Information Security Management System (ISMS) to ensure the protection of the healthcare data it has access to and to meet the requirements of the HIPAA Security Rule. Any data displayed on the TriNetX Platform in aggregate form, or any patient-level data provided in a data set generated by the TriNetX Platform, only contains de-identified data as per the de-identification standard defined in Section §164.514(a) of the HIPAA Privacy Rule. The process by which the data is de-identified is attested to through a formal determination by a qualified expert, as defined in Section §164.514(b) [1] of the HIPAA Privacy Rule. This legal determination by a qualified expert, refreshed in December 2020, supersedes the need for TriNetX's previous waiver from the Western Institutional Review Board (IRB). The TriNetX network contains data provided by participating Healthcare Organizations (HCOs), each of which represents and warrants that it has all necessary rights, consents, approvals, and authority to provide the data to TriNetX under a Business Associate Agreement (BAA), so long as their name remains anonymous as a data source and their data are utilized for research purposes. The data shared through the TriNetX Platform are attenuated to ensure that they do not include sufficient information to determine which HCO contributed specific details on a patient [Available from: https://trinetx.com/trinetx-publication-guidelines/].

## Consent for publication

Not applicable.

## Availability of data and materials

The data that support the findings of this study are available from TriNetX. While restrictions apply to the availability of these data, which were used under license for the current research and are not publicly available, the data are available from the authors upon reasonable request and with the permission of TriNetX.

## Funding

The present study was not funded.

## Declaration of competing interest

The authors declare having no conflict of interest.

## List of Abbreviations

CI: confidence interval
CONSORT: Consolidated Standard of Reporting Trials
HR: Hazard ratio
OR: odds ratio
RR: risk ratio
SMR: standardized mortality ratio
