# Supplementary material for: A propensity score-matched study including 250,000 patients with Factor V Leiden shows significantly increased mortality in comparison with individuals without thrombophilia
Source: Public Health Pract (Oxf). 2026 Feb 16;11:100751. doi: 10.1016/j.puhip.2026.100751 (PMC12926590; doi:10.1016/j.puhip.2026.100751)
Supplement: Multimedia component 1 [file mmc1.docx]

**Supplementary Figure S1**

Modified Consolidated Standard of Reporting Trials (CONSORT) flow chart.

The chart shows the data extraction process from the TriNetX database. The total cohort size of n = 21,020,687 subjects consisted of 241,572 individuals positively tested for Factor V Leiden mutation and 20,779,115 patients without Factor V mutation. The database was accessed on November 19, 2024.

**Supplementary Table S1**

Analysis of the risk of venous thrombosis among the cohorts I (patients with Factor V Leiden mutation) and II (individuals without Factor V Leiden mutation).

| Risk of thrombosis analysis | | | | | | | |
| --- | --- | --- | --- | --- | --- | --- | --- |
| Cohort | Factor V mutation | Patients in cohort | Patients with outcome | Risk | p | Risk ratio (95% CI) | Odds ratio (95% CI) |
| I | + | 241,572 | 61,588 | 25.5% | <0.001 | 7.202 (7.046-7.362) | 9.325 (9.109-9.546) |
| II | - | 241,572 | 8,551 | 3.5% |  |  |  |

**Supplementary Table S2**

Kaplan-Meier survival analysis comparing patients with Factor V Leiden mutation (cohort I) and individuals without Factor V Leiden mutation (cohort II). The time window was 6.935 days. The outcome was death in terms of all-cause mortality.

| Kaplan-Meier survival analysis | | | | | |
| --- | --- | --- | --- | --- | --- |
| Cohort | Factor V mutation | Patients in cohort | Survival probability | p | Hazard ratio (95% CI) |
| I | + | 241.572 | 65,90% | <0,001 | 1,365 (1,341-1,391) |
| II | - | 241.572 | 70,69% |  |  |

Figure S2. In contrast to the findings observed in the female subcohort, age-stratified survival analyses in the male subcohort revealed no significant differences in all-cause mortality between individuals with and without FVL across all age groups.

**Supplementary Table S3**

Analysis of the risk of death and venous thrombosis among the subcohorts III (females with Factor V Leiden mutation) and IV (females without Factor V Leiden mutation).

| Subcohort | Factor V mutation | Patients in subcohort | Patients with outcome | Risk | p | Risk ratio (95% CI) | Odds ratio (95% CI) |
| --- | --- | --- | --- | --- | --- | --- | --- |
| Risk of death analysis | | | | | | | |
| III | + | 143,344 | 13,042 | 9.1% | <0.001 | 1.453 (1.416-1.492) | 1.499 (1.458-1.541) |
| IV | - | 143,344 | 8,973 | 6.3% |  |  |  |
| Risk of thrombosis analysis | | | | | | | |
| III | + | 143,344 | 32,587 | 22.7% | <0.001 | 7.850 (7.607-8.101) | 9.866 (9.543-10.200) |
| IV | - | 143,344 | 4,151 | 2.9% |  |  |  |


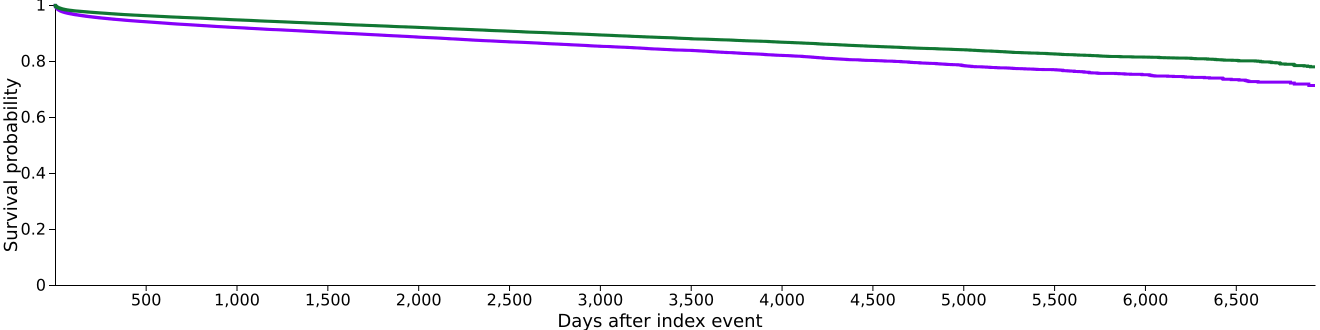


**Supplementary Figure S2**

Kaplan-Meier survival analysis of females positively tested for Factor V Leiden mutation (purple line) and women without Factor V mutation (green line). The survival probability at the end of the time window of 6.935 days was significantly different (71.25% vs. 77.91%; p<0.001; Log-Rank test).

These results are summarized in Supplementary Tables S4–S6 and Supplementary Figures S3–S5.

**Supplementary Table S4**

Analysis of the risk of death among the subcohorts V-X (females with and without Factor V Leiden mutation stratified by age).

| **Risk of death analysis** | | | | | | | | |
| --- | --- | --- | --- | --- | --- | --- | --- | --- |
| Subcohort | Age (years) | Factor V mutation | Patients in subcohort | Patients with outcome | Risk | p | Risk ratio (CI95%) | Odds ratio (CI95%) |
| V | <20 | + | 4,241 | 186 | 4.4% | <0.001 | 9.300 (5.875-14.721) | 9.681 (6.092-15.385) |
| VI | <20 | - | 4,241 | 20 | 0.5% |  |  |  |
| VII | 20-50 | + | 70,494 | 1,668 | 2.4% | <0.001 | 2.313 (2.121-2.523) | 2.345 (2.148-2.561) |
| VIII | 20-50 | - | 70,494 | 721 | 0.1% |  |  |  |
| IX | >50 | + | 72,456 | 11,507 | 15.9% | <0.001 | 1.364 (1.328-1.400) | 1.432 (1.390-1.476) |
| X | >50 | - | 72,456 | 8,439 | 11.6% |  |  |  |

**Supplementary Table S5**

Analysis of the risk of venous thrombosis among the subcohorts V-X (females with and without Factor V Leiden mutation stratified by age).

| **Risk of thrombosis analysis** | | | | | | | | |
| --- | --- | --- | --- | --- | --- | --- | --- | --- |
| Subcohort | Age (years) | Factor V mutation | Patients in subcohort | Patients with outcome | Risk | p | Risk ratio (CI95%) | Odds ratio (CI95%) |
| V | <20 | + | 4,241 | 700 | 16.5% | <0.001 | 53.846 (31.161-93.047) | 64.293 (73.077-111.486) |
| VI | <20 | - | 4,241 | 13 | 0.3% |  |  |  |
| VII | 20-50 | + | 70,494 | 10,945 | 15.5% | <0.001 | 11.910 (11.143-12.729) | 13.915 (12.998-14.897) |
| VIII | 20-50 | - | 70,494 | 919 | 1.3% |  |  |  |
| IX | >50 | + | 72,456 | 21,124 | 29.2% | <0.001 | 6.564 (6.335-6.802) | 8.854 (8.517-9.204) |
| X | >50 | - | 72,456 | 3,218 | 4.4% |  |  |  |

**Supplementary Table S6**

Kaplan-Meier survival analysis of the subcohorts V-X (females with and without Factor V Leiden mutation stratified by age).

| **Kaplan-Meier survival analysis** | | | | | | |
| --- | --- | --- | --- | --- | --- | --- |
| Subcohort | Age (years) | Factor V mutation | Patients in subcohort | Survival probability | p | Hazard ratio (CI95%) |
| V | <20 | + | 4,241 | 89.43% | <0.001 | 7.474 (4.711-11.859) |
| VI | <20 | - | 4,241 | 98.51% |  |  |
| VII | 20-50 | + | 70,494 | 91.41% | <0.001 | 2.182 (1.999-2.381 |
| VIII | 20-50 | - | 70,494 | 95.86% |  |  |
| IX | >50 | + | 72,456 | 59.28% | <0.001 | 1.352 (1.315-1.391) |
| X | >50 | - | 72,456 | 63.95% |  |  |


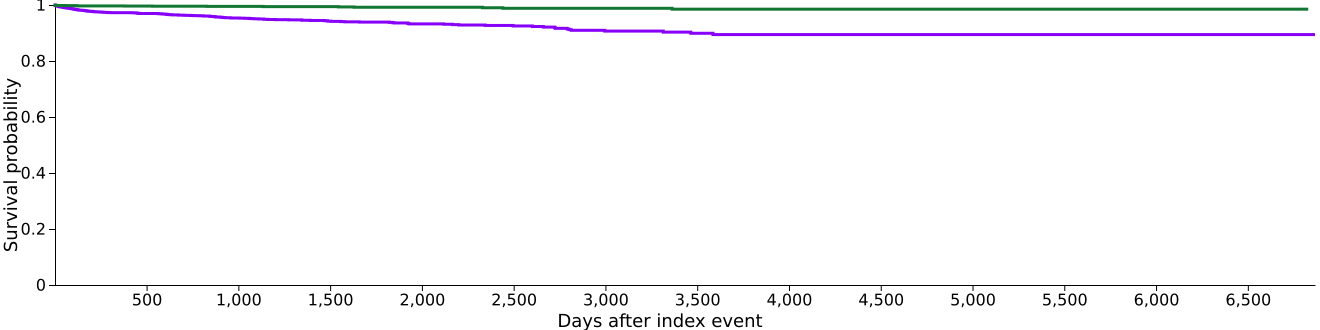


**Supplementary Figure S3**


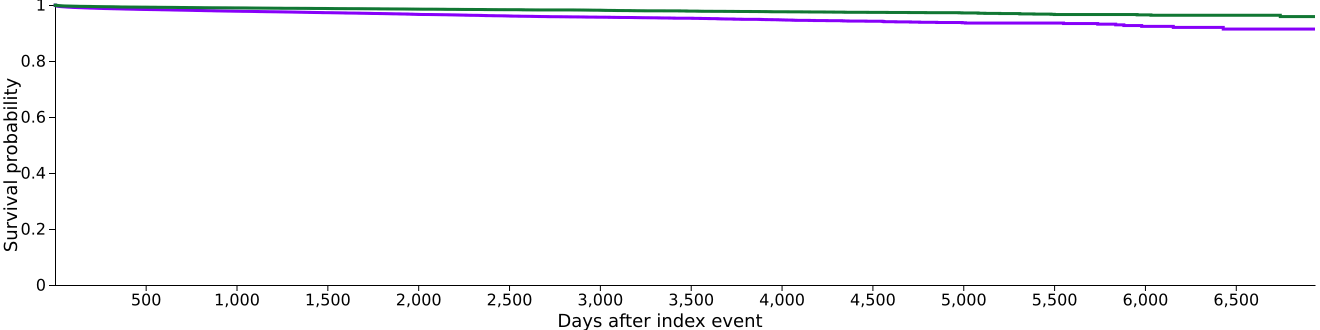


**Supplementary Figure S4**


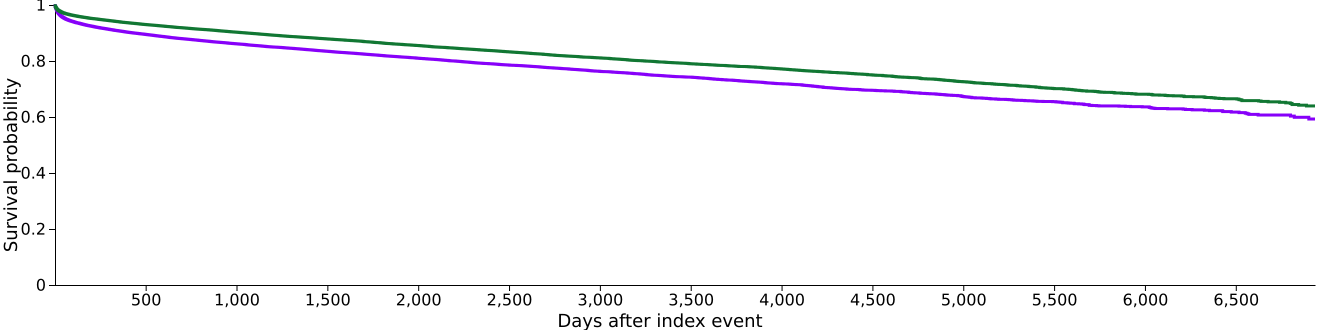


**Supplementary Figure S5**

Supplementary Figures S3-S5

Kaplan-Meier survival analysis of females positively tested for Factor V Leiden mutation (purple line) and women without Factor V mutation (green line). Age strata of <20 (Supplementary Figure S3), 20-50 (Supplementary Figure S4), and >50 years (Supplementary Figure S5) were formed. The respective survival probability at the end of the time window of 6.935 days was significantly different among all subcohorts (p<0.001; Log-Rank test).
